# Serum ferritin and risk of stroke: a meta-analysis of observation studies

**DOI:** 10.3389/fneur.2025.1539407

**Published:** 2025-07-01

**Authors:** Wen Zheng, Yangyang Wang, Zuwei Xia, Dengliang Liu

**Affiliations:** ^1^School of Preclinical Medicine, Zunyi Medical University, Zunyi, China; ^2^Department of Neurosurgery, Sichuan Provincial People’s Hospital, University of Electronic Science and Technology of China, Chengdu, China; ^3^Department of Gastrointestinal Surgery, Chongqing Jiulongpo People's Hospital, Chongqing, China; ^4^Department of General Surgery, Xipeng Town Health Center of Jiulongpo District, Chongqing, China

**Keywords:** serum ferritin, stroke, meta-analysis, cerebrovascular accident, iron

## Abstract

**Background and purpose:**

Serum ferritin, a well-established biomarker of iron status, has been inconsistently linked to stroke risk in previous studies. This meta-analysis aims to systematically evaluate the association between serum ferritin levels and the risk of stroke.

**Methods:**

We conducted a systematic literature search across the PubMed and Embase databases to identify relevant studies. Studies meeting predefined eligibility criteria were selected, and relevant data were extracted. Statistical analysis was performed using Stata 12.0.

**Results:**

Ten studies, including eight longitudinal and three cross-sectional, were included in our meta-analysis. Cross-sectional studies showed that stroke patients had significantly higher serum ferritin levels than controls. Longitudinal studies suggested a 22% increase in stroke risk in individuals with higher serum ferritin. Subgroup analysis indicated that further high-quality population-based cohort studies are warranted to validate these findings. Dose–response meta-analysis confirmed a positive association between serum ferritin levels and stroke risk.

**Conclusion:**

This meta-analysis provides evidence of a positive association between increased serum ferritin levels and stroke incidence. While these results are promising, definitive conclusions cannot be drawn at this time. Therefore, additional robust, prospective cohort studies are imperative to substantiate this relationship.

## Introduction

Stroke is a serious cerebrovascular disease. Current data indicates that approximately 12 million people suffer strokes annually, making it one of the most lethal diseases worldwide (1, 2). Although advances in active thrombolytic therapy and surgical interventions have substantially decreased stroke-related mortality, the persistently high incidence of disability and long-term sequelae renders stroke a profoundly debilitating disorder. As a result, preventive strategies have emerged as a paramount focus in stroke management.

Iron is a vital element for human health; however, high concentrations of iron can induce severe cytotoxicity via the Fenton reaction. Furthermore, iron status is closely linked to cardiovascular disease (3–7). Serum ferritin, a key indicator of iron storage/iron level, indirectly reflects whether an individual is experiencing iron deficiency or overload (8–10). This suggests that serum ferritin levels can serve as a proxy for overall health to some extent. However, observational studies have yielded inconsistent findings regarding the association between serum ferritin and stroke risk (11–19). To clarify this relationship, a meta-analysis was conducted aimed at evaluating the quantitative association between serum ferritin levels and the risk of stroke.

## Methods

The review was conducted in accordance with the PRISMA guidelines and was prospectively registered with the PROSPERO database of systematic reviews (CRD42024628065).

### Literature search

Literature concerning ferritin and stroke was systematically searched using the PubMed and Embase databases. Two thematic categories were combined with the Boolean operator “AND”. The search incorporated the following terms: Ferritins, Iron, Stroke, Cerebrovascular Disorders, Cerebral Hemorrhage, Cerebral Infarction, Subarachnoid Hemorrhage.

### Inclusion and exclusion criteria

Literature was included as the following criteria: (1) Studies were included if they provided data on or allowed for the estimation of the association between serum ferritin levels and stroke. (2) Studies were included if they reported risk ratios (RRs) or odds ratios (ORs) with their corresponding 95% confidence intervals (CIs), or if these could be calculated from the reported data. (3) In the longitudinal studies, serum ferritin levels were at minimum categorized into high-level and low-level groups. Literature met following criteria were excluded: (1) Meeting abstracts were excluded due to insufficient data available online. (2) In cases where studies utilized the same participant population cohort, the study with the largest sample size was included, and other studies from this population cohort were excluded. (3) Studies were excluded if they presented stroke data as part of total cardiovascular disease data, without providing sufficient detailed information specific to stroke.

### Literature quality assessment

Newcastle–Ottawa Quality Assessment Scale (NOS) was used to assess the literature quality. Literature with score equal to 9 were regarded as high quality, otherwise, a score lower than 9 is low quality. The detailed data are supplied in Supplementary Table S1.

### Data abstraction

Data were independently extracted by two authors, Zheng and Wang. Any disagreements were resolved through discussion. The data extraction contents contain the following points: The first author’s name, year of publication, sample size, number of cases, mean age at baseline, time of mean follow up, adjusted factors, type of stroke, gender, serum ferritin level and the RRs/ORs. Note on Serum Ferritin Levels: When serum ferritin levels were reported as a range, the average value was estimated assuming a normal distribution.

### Statistical analysis

Stata 12.0 was used as statistical analysis software. A fixed effects model was the prior, if met I^2^ > 50%, indicating a large heterogeneity, use the random effect model instead. Then category analysis was performed by the characteristics (Quality of original literature, adjust risk factors, Sex, mean age at baseline, Race) provides in these literatures. For dose response meta-analysis, linear and a cubic spline model were both used to assess the relationship between serum ferritin and stroke risk. And sensitivity analysis was also used to evaluate the reliability of our results. Due to the results are positive or not often affects the publication of the literature, then the Begg test was used for assessment of publication bias.

Statistical analyses were performed using Stata 12.0. A fixed-effects model was initially employed for meta-analysis. However, if significant heterogeneity was detected (I^2^ > 50%), a random-effects model was used instead. Subgroup analyses were conducted based on characteristics reported in the included studies, such as study quality, adjusted risk factors, sex, mean age at baseline, and race. For dose–response meta-analysis, a linear and a cubic spline model were applied to investigate the relationship between serum ferritin levels and stroke risk. Sensitivity analyses were performed to evaluate the reliability of our findings. Publication bias was assessed using Begg’s test and Egger’s test, given that the likelihood of publication can be influenced by the direction of the results.

## Results

### Literature search and study characteristics

**Figure 1:**
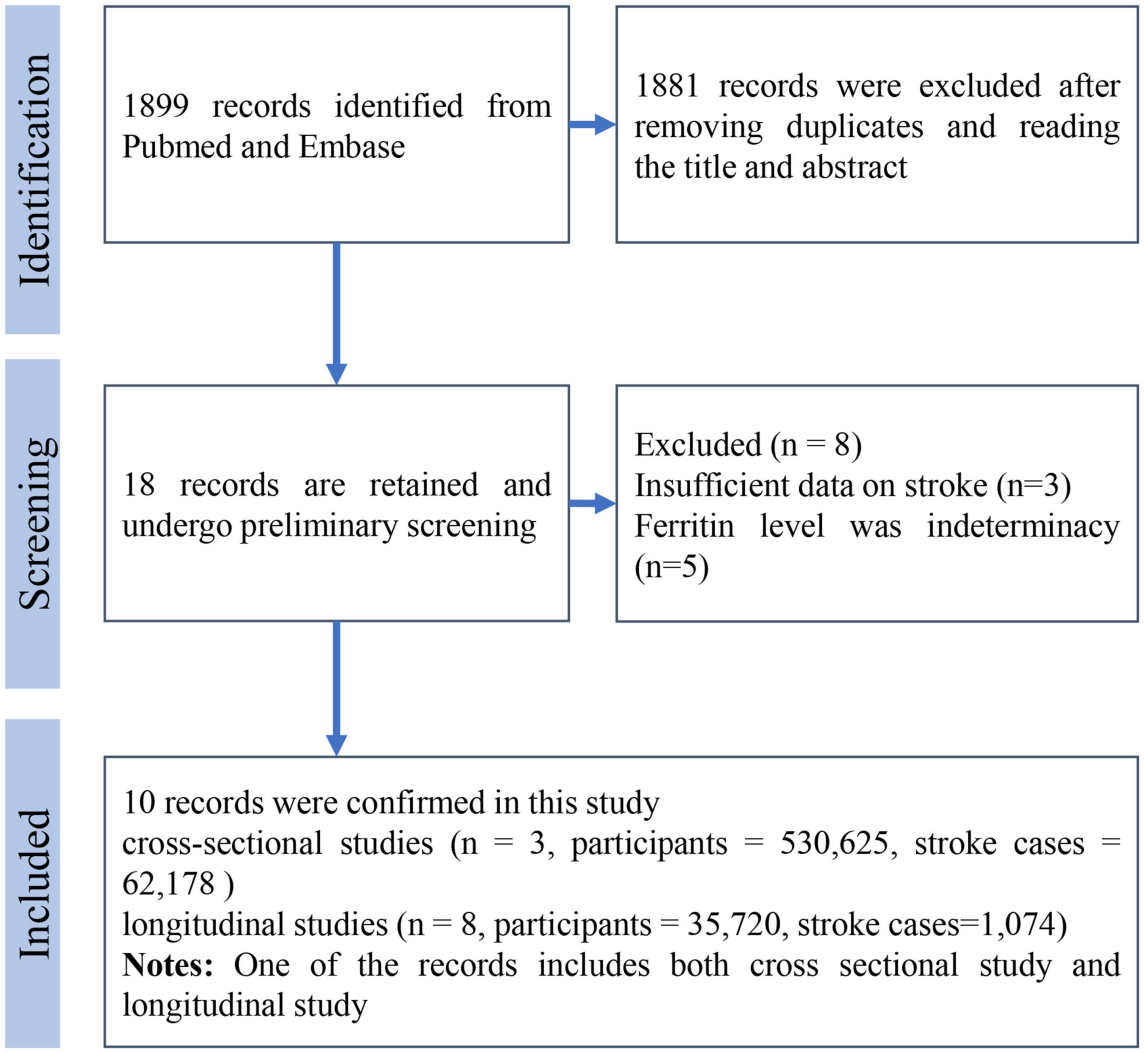
Flow chart of literature retrieval and screening.

The literature search process is illustrated in Figure 1. Initially, 1899 records were identified through database searching. After two rounds of meticulous screening, 10 literatures were ultimately included in the meta-analysis (11–18, 20, 21). Among these literatures, three were cross-sectional studies and eight were longitudinal studies. The cross-sectional studies involved 530,625 participants, while the longitudinal studies included 35,720 participants. In the cross-sectional studies, there were 62,178 stroke patients, although one study did not report the number of stroke cases. For longitudinal studies, 1,074 cases were reported. The mean age of participants at baseline ranged from 41 to 64.8 years. The mean follow-up time for longitudinal studies varied from 4.3 to 17 years. Seven studies were conducted in Europe, two in North America, and one in Asia. All detailed information is summarized in Table 1.

**Table 1:** Characteristics of studies of serum ferritin and the risk of stroke.

Author (country)	Year of publication	character of study	Data source	Sample size	No. of total stroke cases (type)	Sex	Location	Mean age at baseline	Mean follow-up years	Adjustment factors
M. P. Hermans et al. (18)	2010	*Cross-sectional study*	Not Reported	424	16	Only man	Europe	64.8	Not Applicable	Not adjust
Dipender Gill et al. (16)	2018	*Cross-sectional study*	Not Reported	521,612	652	Men and woman	Europe	Not Reported	Not Applicable	Not adjust
Dong Liu et al. (19)	2021	*Cross-sectional study* & longitudinal study	China Health and Nutrition Survey	8,589 7,290	Not Reported 128	Men and woman	Asia	Not Reported 51	Not Applicable 6.1	Adjusted for age, gender, BMI, smoking status, drinking status, diabetes, hypertension, and hsCRP
James S. Pankow et al. (17)	2008	Longitudinal study	Atherosclerosis Risk in Communities (ARIC) Study	6,813	245	Men and woman	North America	54.4	15	Not adjust
Vander et al. (11)	2005	Longitudinal study	European Prospective Investigation Into Cancer and Nutrition (EPIC)	1,132	65	Only women	Europe	59	4.3	Adjusted for age, body mass index, alcohol intake, hsCRP, smoking, hypertension, hypercholesterolemia, diabetes, glucose, LDL cholesterol, and HDL-cholesterol
Sotirios Tsimikas et al. (14)	2009	Longitudinal study	Bruneck study cohort	765	Not Reported	Men and woman	Europe	62.7	10	Adjusted for age and sex
Knuiman MW et al. (13)	2003	Longitudinal study	Busselton Health Survey	1,612	118	Men and woman	Oceania	58.5	17	After adjustment for age and other cardiovascular disease risk factors
Kim Ekblom et al. (15)	2006	Longitudinal study	Västerbotten Intervention Project	6,952	231	Men and woman	Europe	54.9	13	Adjusted for BMI, hypertension, smoking, diabetes, cholesterol, hsCRP, HFE C282Y and HFE H63D.
Obiora Egbuche et al. (20)	2017	Longitudinal study	Jackson Heart Study	4,659	137	Men and woman	North America	54	8	Adjusted for age, sex, body mass index, smoking status, alcohol use within past 12 months, physical activity score, systolic blood pressure, antihypertensive medication use, low density lipoprotein cholesterol, logarithm of triglycerides, lipid lowering medication use status, fatty liver disease, income, and education
Milton Fabian et al. (12)	2022	Longitudinal study	Scottish health surveys	6,497	150	Men and woman	Europe	41	14.1	Adjusted for age, sex/menopausal status, fibrinogen levels, GGT levels, alcohol intake, smoking, systolic blood pressure, diastolic blood pressure, total cholesterol, HDL cholesterol, body mass index and year of survey

### Results of qualitative analysis

**Figure 2:**
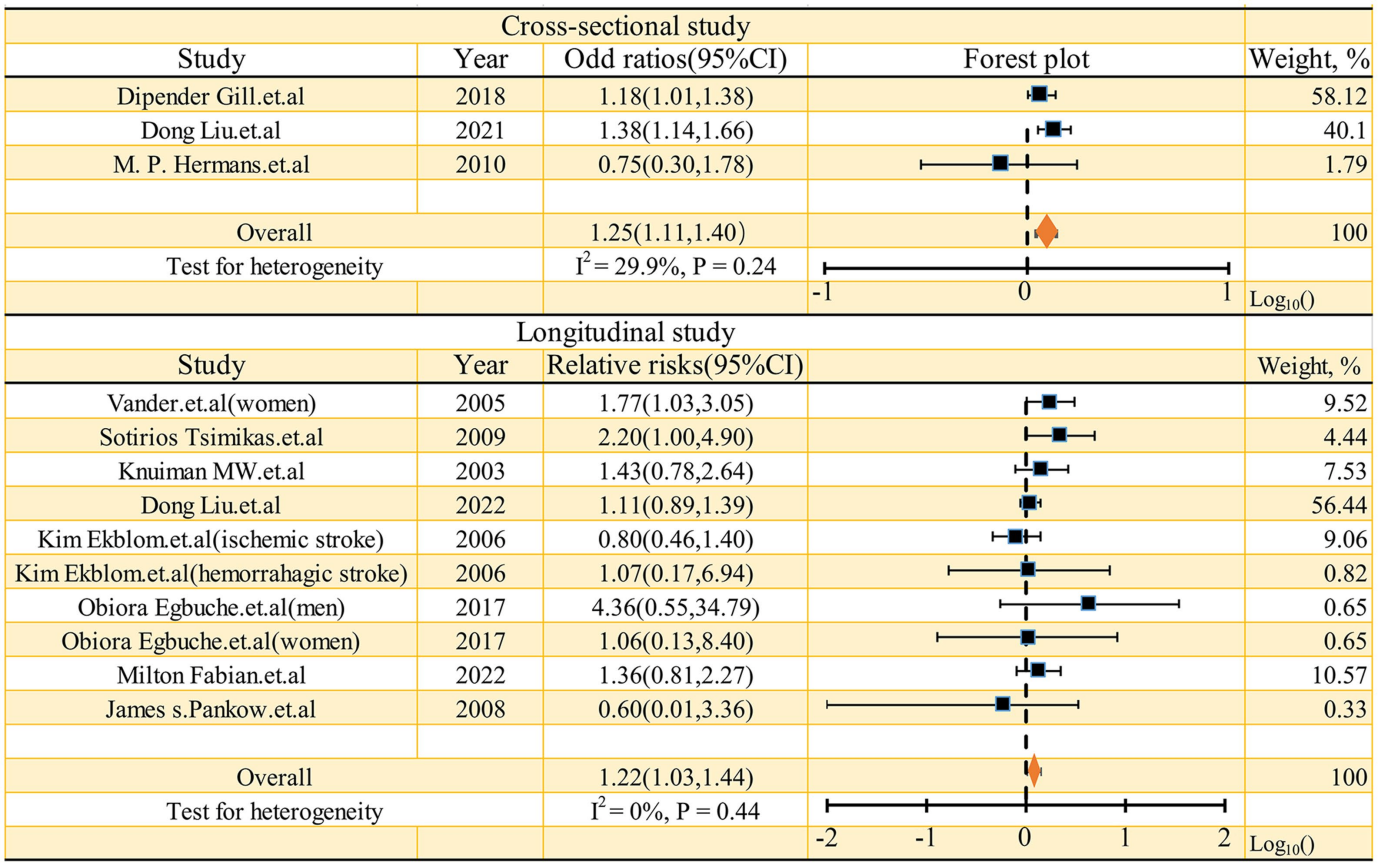
Forest plot of studies examining the association between serum ferritin and risk of stroke.

**Figure 3:**
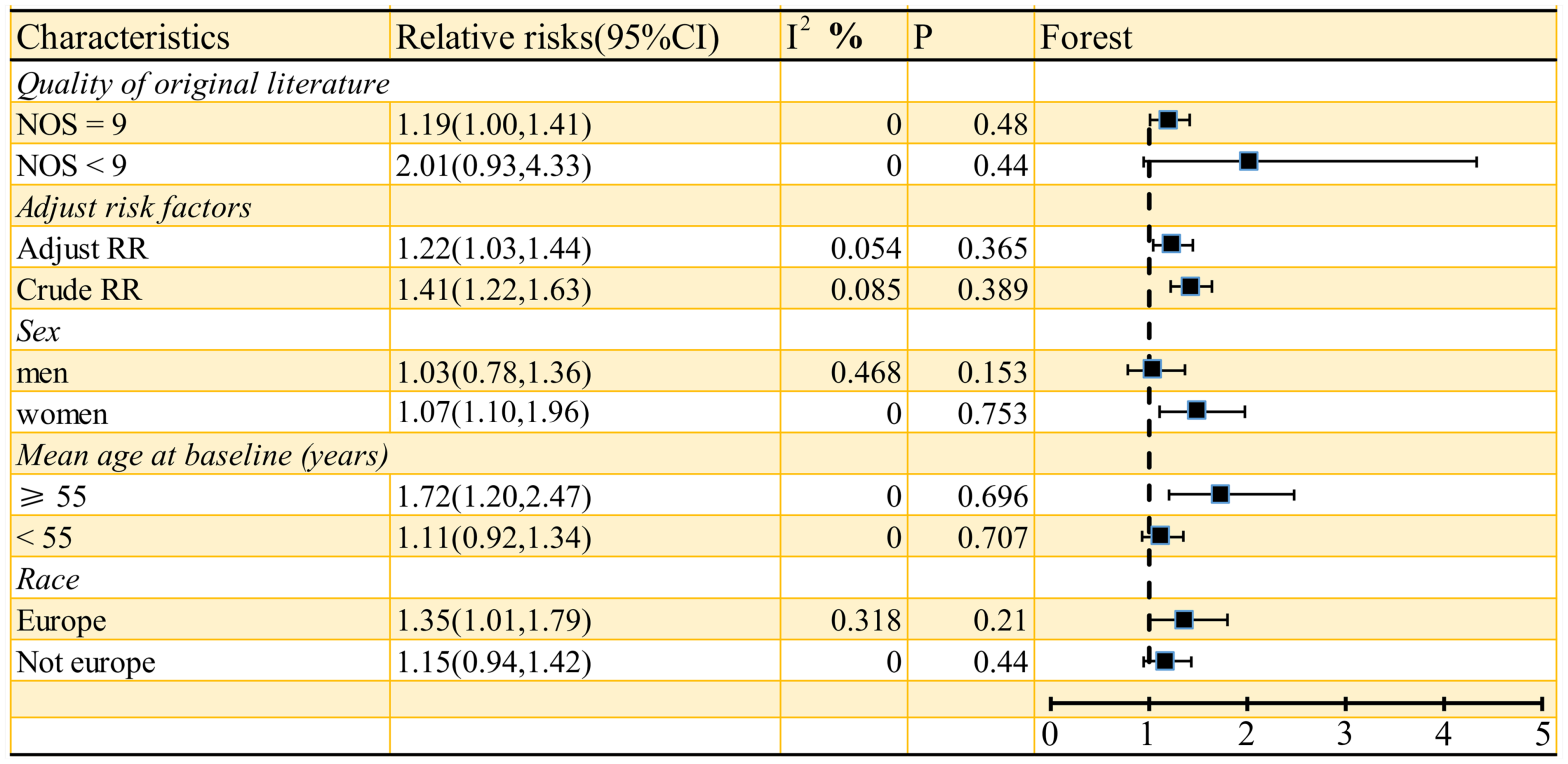
Subgroup analyses between serum ferritin and risk of stroke for longitudinal studies.

**Figure 4:**
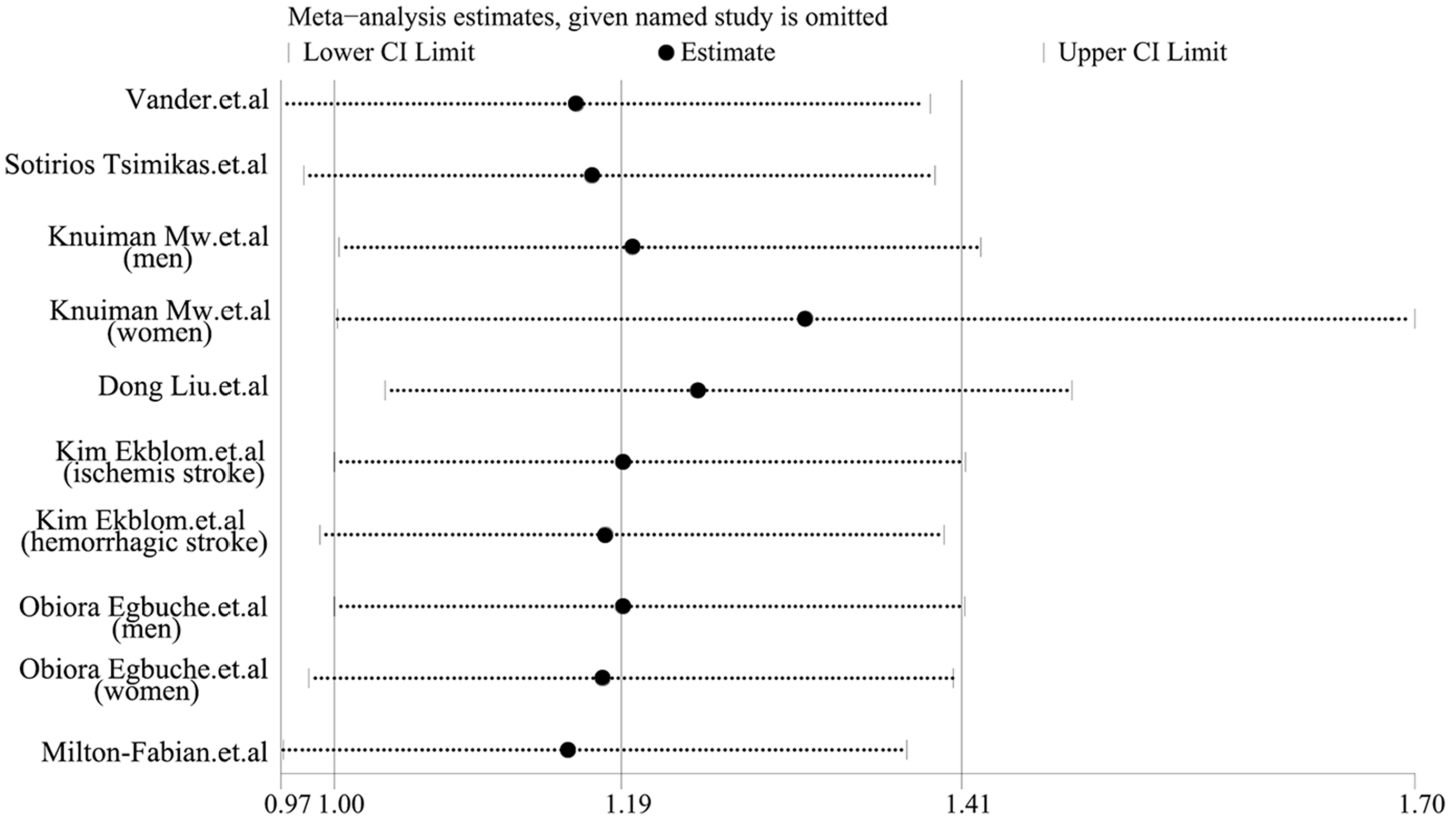
Sensitivity analysis of serum ferritin and stroke risk for longitudinal study.

As shown in Figure 2, the pooled odds ratio (OR) for cross-sectional studies was 1.25 (95% CI: 1.11, 1.40), with low heterogeneity (I^2^ = 29.9%, *p* = 0.24). A qualitative analysis of longitudinal studies indicated a 22% increased risk of stroke incidence in individuals with high serum ferritin levels (RR = 1.22, 95% CI: 1.03–1.44, I^2^ = 0%, *p* = 0.44). Further categorical analyses of longitudinal studies, stratified by study characteristics (quality, adjustment for risk factors, sex, mean age at baseline, and race), yielded inconsistent results, as detailed in Figure 3. Sensitivity analysis (Figure 4) revealed that excluding certain studies resulted in non-significant findings.

### Dose response meta-analysis

**Figure 5:**
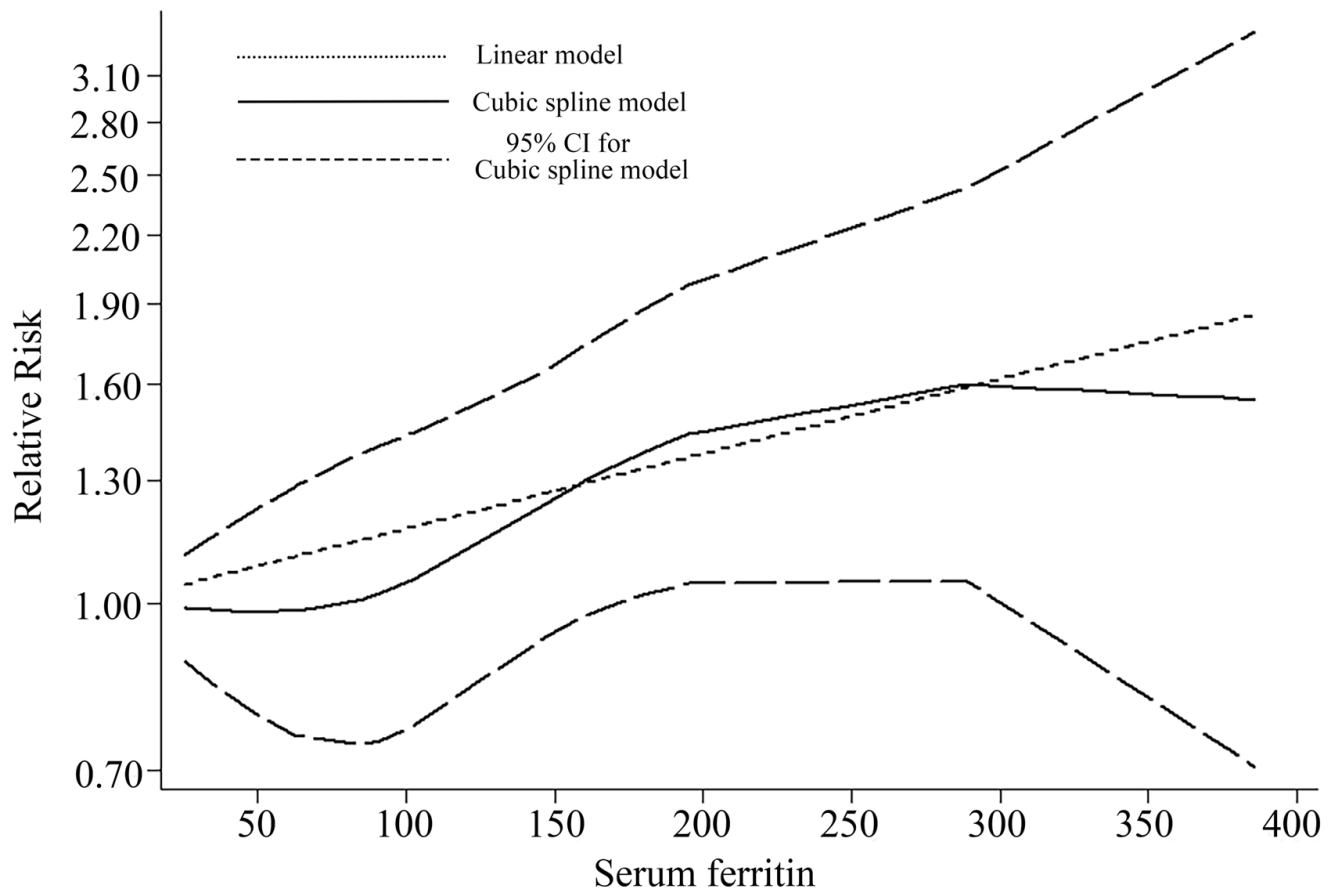
Dose–response for relationship between serum ferritin and risk of stroke by linear and a cubic spline model.

Based on the data provided in the referenced literature, four studies (11–13, 20) were utilized for dose–response meta-analysis. Figure 5 illustrates the quantitative results from longitudinal studies. The cubic spline model analysis suggests an approximate positive correlation between serum ferritin levels and the incidence of stroke (*p* = 0.1053). The linear quantitative model analysis further revealed that for every 50 ng/mL increase in serum ferritin, the risk of stroke increases by 8% (RR = 1.08, 95% CI 1.01–1.16, *p* = 0.02). Specifically: A 100 ng/mL increase in serum ferritin corresponds to a 17% increased risk of stroke (RR = 1.17, 95% CI 1.02–1.35); A 150 ng/mL increase corresponds to a 27% increased risk (RR = 1.27, 95% CI 1.03–1.57); A 200 ng/mL increase corresponds to a 38% increased risk (RR = 1.38, 95% CI 1.04–1.83). These findings underscore the potential stroke risk associated with elevated serum ferritin levels and advocate for monitoring and management strategies in clinical settings.

### Publication bias

The Begg’s test (*p* = 0.353 > 0.05) and Egger’s test (*p* = 0.248 > 0.05) found no obvious publication bias for this meta-analysis (Supplementary Figure S1).

## Discussion

As we know, this is the first time that we have evaluated the relationship between serum ferritin and stroke risk through meta-analysis. In this meta-analysis, the results indicated that individuals with high serum ferritin levels may have a higher risk of stroke compared to those with low serum ferritin levels. Furthermore, a dose–response meta-analysis also revealed a positive correlation between serum ferritin and stroke risk. Additionally, the meta-analysis of cross-sectional studies suggested that there may be a close association between serum ferritin and stroke.

Several potential mechanisms underlie this phenomenon. Firstly, numerous clinical studies have demonstrated that serum ferritin levels are closely associated with carotid atherosclerosis, hypertension, diabetes, steatotic liver and other conditions, these factors have been established as risk factors for stroke onset. Therefore, we hypothesize that serum ferritin may indirectly reflect stroke occurrence through its association with carotid atherosclerosis, hypertension, diabetes, steatotic liver and other conditions (22–26). Secondly, as a critical indicator of iron homeostasis, serum ferritin levels are markedly elevated in the context of pathological iron excess. Iron overload can generate reactive oxygen species via the Fenton reaction (27, 28), further triggering inflammatory responses, damaging vascular endothelial cells, and increasing the risk of cardiovascular and cerebrovascular disease (29). Additionally, research has shown that high red meat consumption is linked to cardiovascular diseases (30), and individuals with higher red meat intake typically have elevated serum ferritin levels (31).

Previous meta-analyses have suggested a positive association between ferritin levels and the risk of coronary artery disease (32), given the shared pathophysiology between coronary artery disease and stroke to some extent (As mentioned previously, diseases such as carotid atherosclerosis, hypertension, diabetes, steatotic liver and other conditions), this finding further implies that serum ferritin could serve as a predictive risk factor for stroke occurrence. In our meta-analysis, we pooled three cross-sectional studies, yielding a pooled odds ratio (OR) of 1.25 (95% CI: 1.11, 1.40), with a low level of heterogeneity (I^2^ = 29.9%, *p* = 0.24). While cross-sectional studies cannot establish causality, they do suggest a close relationship between serum ferritin levels and stroke. Moreover, the longitudinal studies included in our meta-analysis offer more robust evidence supporting the role of serum ferritin as a predictive risk factor. Meta-analysis of these longitudinal studies demonstrated a positive correlation between serum ferritin levels and stroke risk (high level serum ferritin vs. low level ferritin, RR = 1.22, 95% CI: 1.03–1.44, I^2^ = 0, *p* = 0.44). Consequently, we have reason to believe that serum ferritin can be considered as a valuable indicator for predicting the occurrence of stroke.

While this meta-analysis yielded a positive overall result, certain limitations must be acknowledged. Although the total qualitative analysis was positive, the categorical analysis (based on the quality of original literature, adjustment of risk factors, sex, mean age at baseline, and race) for longitudinal studies revealed several negative findings (Figure 3). Moreover, the sensitivity analysis also produced some negative results. These findings suggest that the robustness of our results should be carefully reconsidered, despite the possibility that the negative results stem from the limited number of studies included in the subgroup analysis. Second, we used the highest and lowest serum ferritin groups in the article to conduct the qualitative analysis, but in some literature, the exact serum ferritin level is indeed unclear (14, 15, 19), potentially contributing to a false positive result. Thirdly, according to the cubic spline model dose–response meta-analysis results, the population with extremely low serum ferritin levels (within the 0 to 100 μg/L range) demonstrated a little higher incidence of stroke. Although this finding did not reach statistical significance, several potential explanations warrant consideration. Supporting this, some studies have identified excessively low serum ferritin as a potential risk factor for stroke (11), and a link between iron-deficiency anemia and increased stroke risk has also been reported (33, 34). Therefore, more high-quality cohort studies are needed to determine whether excessively low serum ferritin levels can be used as a reliable predictor of stroke.

## Conclusion

This meta-analysis revealed a positive correlation trend between serum ferritin levels and stroke risk. However, the categorical analysis and sensitivity analysis provided less conclusive results. Therefore, further studies are needed to validate and strengthen our findings.

## Data Availability

The original contributions presented in the study are included in the article/Supplementary material, further inquiries can be directed to the corresponding authors.
