# Supplementary material for: Serum ferritin and risk of stroke: a meta-analysis of observation studies
Source: Front Neurol. 2025 Jul 1;16:1539407. doi: 10.3389/fneur.2025.1539407 (PMC12259440; doi:10.3389/fneur.2025.1539407)
Supplement: Supplementary file 1 [file Data_Sheet_1.docx]

**Serum ferritin and risk of stroke: a meta‑analysis of observation studies**

Authors: Wen Zheng, BS ^1 a^, Yangyang Wang, PhD ^2 a^, Zuwei Xia, MD^3^, Dengliang Liu, MD ^3,4^.

^1^ School of Preclinical Medicine of Zunyi Medical University, Zunyi, 563003, China

^2^ Department of Neurosurgery, Sichuan provincial People’s Hospital, University of Electronic Science and Technology of China, Chengdu, 611731, China

^3^ Department of Gastrointestinal Surgery, Chongqing Jiulongpo People's Hospital, Chongqing, China

^4^ Department of General Surgery, Xipeng Town Health Center of Jiulongpo District, Chongqing, China

^a^ These authors contributed equally to this work and share first authorship

Address of email

Correspondence author:

Zuwei Xia; 935307333@qq.com

Dengliang liu; 13340210482@163.com

Co-author:

Wen Zheng, E-mail: [2086139535@qq.com](mailto:916213794@qq.com);

Yangyang Wang, E-mail: 1476806473@qq.com;

Literature retrieval strategy

PubMed:

#1."Ferritins"[Mesh] "Iron"[Mesh]

#2."Stroke"[Mesh]

#3."Cerebrovascular Disorders"[Mesh]

#4."Cerebral Hemorrhage"[Mesh]

#5."Cerebral Infarction"[Mesh] OR "Brain Ischemia"[Mesh]

#6."Subarachnoid Hemorrhage"[Mesh]

(((("Subarachnoid Hemorrhage"[Mesh]) OR ("Cerebral Infarction"[Mesh] OR "Brain Ischemia"[Mesh])) OR ("Cerebral Hemorrhage"[Mesh])) OR ("Cerebrovascular Disorders"[Mesh])) OR ("Stroke"[Mesh])

("Iron"[Mesh]) OR ("Ferritins"[Mesh])

(("Iron"[Mesh]) OR ("Ferritins"[Mesh])) AND ((((("Subarachnoid Hemorrhage"[Mesh]) OR ("Cerebral Infarction"[Mesh] OR "Brain Ischemia"[Mesh])) OR ("Cerebral Hemorrhage"[Mesh])) OR ("Cerebrovascular Disorders"[Mesh])) OR ("Stroke"[Mesh]))

Covid 19[Title]

((("Iron"[Mesh]) OR ("Ferritins"[Mesh])) AND ((((("Subarachnoid Hemorrhage"[Mesh]) OR ("Cerebral Infarction"[Mesh] OR "Brain Ischemia"[Mesh])) OR ("Cerebral Hemorrhage"[Mesh])) OR ("Cerebrovascular Disorders"[Mesh])) OR ("Stroke"[Mesh]))) NOT (Covid 19[Title])

COVID-19[Title/Abstract]

((("Iron"[Mesh]) OR ("Ferritins"[Mesh])) AND ((((("Subarachnoid Hemorrhage"[Mesh]) OR ("Cerebral Infarction"[Mesh] OR "Brain Ischemia"[Mesh])) OR ("Cerebral Hemorrhage"[Mesh])) OR ("Cerebrovascular Disorders"[Mesh])) OR ("Stroke"[Mesh]))) NOT (COVID-19[Title/Abstract])

((("Iron"[Mesh]) OR ("Ferritins"[Mesh])) AND ((((("Subarachnoid Hemorrhage"[Mesh]) OR ("Cerebral Infarction"[Mesh] OR "Brain Ischemia"[Mesh])) OR ("Cerebral Hemorrhage"[Mesh])) OR ("Cerebrovascular Disorders"[Mesh])) OR ("Stroke"[Mesh]))) NOT (COVID-19[Title/Abstract])

Embase:

#11. #7 AND #8 AND [humans]/lim AND [english]/lim

#10. #7 AND #8 AND [humans]/lim

#9. #7 AND #8

#8. #2 OR #3

#7. #1 OR #4 OR #5 OR #6

#6. 'subarachnoid hemorrhage'/exp OR 'subarachnoid hemorrhage'

#5. 'brain hemorrhage'/exp OR 'brain hemorrhage'

#4. 'brain ischemia'/exp OR 'brain ischemia'

#3. 'iron storage'/exp OR 'iron storage'

#2. 'ferritin'/exp OR 'ferritin'

#1. 'cerebrovascular accident'/exp OR

|  | Representativeness of exposed cohort | Selection of non-exposed cohort | Ascertainment of exposure | Outcome present at start of study | Study controls for age | Study controls for any additional important factor | Assessment of outcome | Length of follow-up>=5 | Adequacy of follow-up<=10% | Score |
| --- | --- | --- | --- | --- | --- | --- | --- | --- | --- | --- |
| Vander.et.al | ☆ | ☆ | ☆ | ☆ | ☆ | ☆ | ☆ | ☆ | ☆ | 9 |
| Sotirios Tsimikas.et.al | ☆ | ☆ | ☆ | / | ☆ | / | ☆ | ☆ | ☆ | 7 |
| JAMES S. PANKOW.et.al | / | ☆ | ☆ | ☆ | ☆ | / | ☆ | ☆ | ☆ | 7 |
| Knuiman MW.et.al | ☆ | ☆ | ☆ | ☆ | ☆ | ☆ | ☆ | ☆ | ☆ | 9 |
| Dipender Gill.et.al | / | ☆ | ☆ | ☆ | / | / | ☆ | / | ☆ | 5 |
| Dong Liu.et.al | ☆ | ☆ | ☆ | ☆ | ☆ | ☆ | ☆ | ☆ | ☆ | 9 |
| M. P. Hermans.et.al | / | ☆ | ☆ | ☆ | ☆ | / | ☆ | ☆ | ☆ | 7 |
| Kim Ekblom.et.al | ☆ | ☆ | ☆ | ☆ | ☆ | ☆ | ☆ | ☆ | ☆ | 9 |
| Obiora Egbuche.et.al | ☆ | ☆ | ☆ | ☆ | ☆ | ☆ | ☆ | ☆ | ☆ | 9 |
| Milton‑Fabian.e | ☆ | ☆ | ☆ | ☆ | ☆ | ☆ | ☆ | ☆ | ☆ | 9 |

Table S1. Literature quality score by Newcastle–Ottawa Scale (NOS) assessment.

|  |
| --- |
| 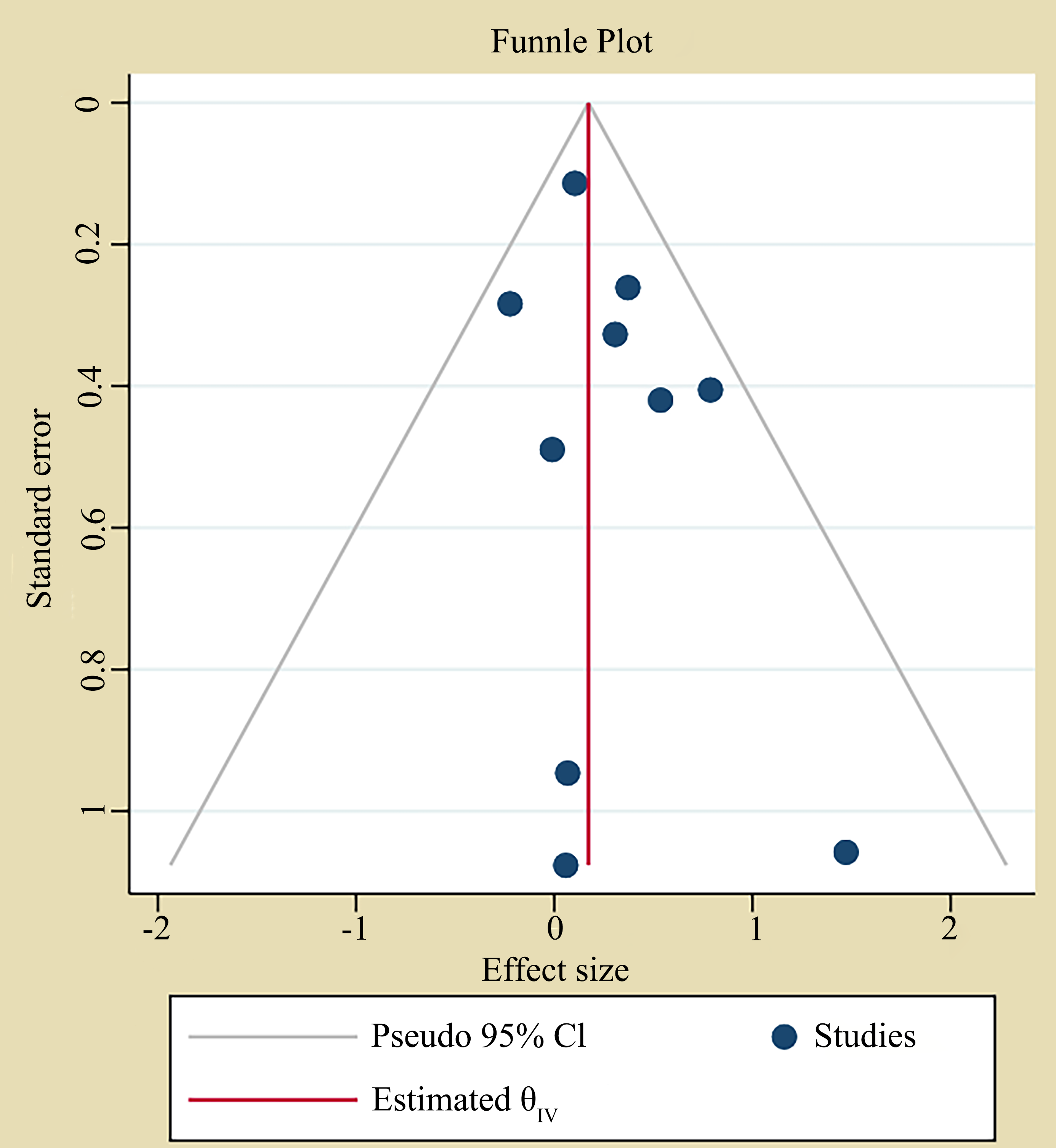  Figure **S1**. Begg test result for literature publication bias. |
